# Wrinkled silica/titania nanoparticles with tunable interwrinkle distances for efficient utilization of photons in dye-sensitized solar cells

**DOI:** 10.1038/srep30829

**Published:** 2016-08-04

**Authors:** Jin Soo Kang, Joohyun Lim, Won-Yeop Rho, Jin Kim, Doo-Sik Moon, Juwon Jeong, Dongwook Jung, Jung-Woo Choi, Jin-Kyu Lee, Yung-Eun Sung

**Affiliations:** 1 Center for Nanoparticle Research, Institute for Basic Science (IBS), Seoul 151-742, Republic of Korea; 2 School of Chemical and Biological Engineering, Seoul National University, Seoul 151-742, Republic of Korea; 3 Department of Chemistry, Seoul National University, Seoul 151-742, Republic of Korea; 4 School of Semiconductor and Chemical Engineering, Chonbuk National University, Jeonju, 561-756, Republic of Korea

## Abstract

Efficient light harvesting is essential for the realization of high energy conversion efficiency in dye-sensitized solar cells (DSCs). State-of-the-art mesoporous TiO_2_ photoanodes fall short for collection of long-wavelength visible light photons, and thus there have been efforts on introduction of scattering nanoparticles. Herein, we report the synthesis of wrinkled silica/titania nanoparticles with tunable interwrinkle distances as scattering materials for enhanced light harvesting in DSCs. These particles with more than 20 times larger specific surface area (>400 m^2^/g) compared to the spherical scattering particles (<20 m^2^/g) of the similar sizes gave rise to the dye-loading amounts, causing significant improvements in photocurrent density and efficiency. Moreover, dependence of spectral scattering properties of wrinkled particles on interwrinkle distances, which was originated from difference in overall refractive indices, was observed.

Solar-to-electric energy conversion has been considered as one of the most promising candidate to replace the conventional energy production based on fossil fuels. Among various kinds of photovoltaic systems, Si and thin film solar cells stand out in the market these days due to their remarkable performance and long-term stability. However, high manufacturing cost hinders their large-scale commercialization, and therefore many other types of photovoltaic devices based on low-cost organic or inorganic materials are under intensive investigations. For a couple of decades, dye-sensitized solar cells (DSCs) have received great attention due to their excellent performance and reliability^1^,^2^,^3^,^4^,^5^,^6^,^7^. Generally, DSCs are based on dye-adsorbed TiO_2_ photoelectrode, which is composed of TiO_2_ nanoparticles of around 20 nm size. The large surface area resulting from this nanostructure is the key for the high performance of DSCs, because it allows large amount of dye-loading and enables enhanced light harvesting. However, the 20 nm dimension which cause negligible amount of scattering in visible light wavelength region leaves considerable portion of incident photons to pass through the photoelectrode without contribution to the solar-to-electric energy conversion^8^,^9^,^10^,^11^.

In addition to the numerous efforts for the development of sensitizers with high extinction coefficient^7^,^12^,^13^,^14^,^15^,^16^,^17^,^18^,^19^,^20^,^21^ and semiconductor oxide electrodes with effective nanostructures^22^,^23^,^24^,^25^,^26^,^27^,^28^,^29^,^30^,^31^, there have been many reports on light scattering materials designed for DSCs in order to maximize the incident light utilization^9^,^10^,^11^,^32^,^33^,^34^,^35^,^36^,^37^,^38^,^39^,^40^,^41^,^42^,^43^,^44^,^45^,^46^,^47^,^48^,^49^,^50^,^51^. Generally, structures with a dimension that is comparable to the wavelength of incident light are known to be efficient for the scattering of photons^10^. Therefore, a few hundreds of nanometer sized scatterers such as cavities and photonic crystals were often employed^32^,^33^,^34^,^35^,^36^,^37^, and TiO_2_ nanoparticles with similar dimension have generally manifested the finest performances^38^,^39^,^40^,^41^,^42^,^43^,^44^,^45^,^46^,^47^,^48^,^49^. These light scattering TiO_2_ particles were incorporated within the photoelectrode by embedding them into the mesoscopic TiO_2_ layer^38^,^39^,^40^ or by forming a layer composed of light scatterers^41^,^42^,^43^,^44^,^45^,^46^,^47^. However, since these scattering particles have insufficient surface area for large amount of dye-loading and corresponding photon absorption, various attempts to increase the surface area of the scattering particles were made^39^,^43^,^44^,^45^,^46^,^47^. Also, there were approaches to tune the scattering properties of the TiO_2_ scattering particles by compositional modifications, and silica/titania core/shell nanoparticles showed fascinating results due to the enhanced light scattering property compared to bare TiO_2_, because of the difference in refractive indices between silica and titania^48^,^49^. Furthermore, aerogel based silica/titania photoanodes and size controlled silica/titania scatterers with enhanced light harvesting properties for DSCs also have been reported^50^,^51^.

Among various types of nanostructured silica materials, wrinkled silica nanoparticles have drawn large interests due to the usefulness of their fibrous morphology and capability for application in numerous fields^52^,^53^,^54^,^55^,^56^,^57^. The formation mechanism of wrinkled morphology was explained by introducing bicontinuous microemulsion phase (Winsor III system)^58^,^59^,^60^, and the methods to control the structure of wrinkles within the nanoparticles were also developed on the basis of understandings on the mechanism. Inspired by the large surface area and ease of morphology control during the synthesis, we herein report wrinkled silica/titania nanoparticles as scattering materials in DSCs, as displayed in Fig. 1. In contrast to the previous reports, wherein alcohols with various alkyl groups were added in order to widen the interwrinkle distances, in this study, wrinkled silica nanoparticles with tunable interwrinkle distances were synthesized by varying the amount of co-solvent (1-pentanol). This quantitative method enabled highly reliable control over the morphologies of wrinkled particles without altering the specific surface area. After coating the particles with TiO_2_ shell, the fabricated wrinkled silica/titania nanoparticles were incorporated into the photoanode of DSCs for efficient light absorption and scattering. In addition to the enhancement in photocurrent density and energy conversion efficiency, further observations on the dependence of spectral scattering properties on interwrinkle distances, of which effectiveness was verified in device applications, were made.

## Results

### Synthesis and morphology control

**Figure 2:**
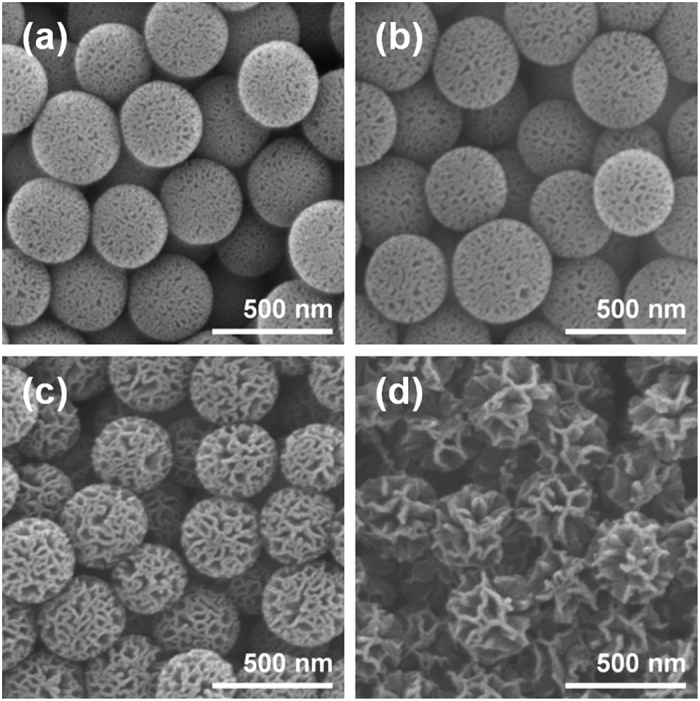
SEM images of wrinkled silica nanoparticles with different interwrinkle distances controlled by the amount of added co-solvent (1-pentanol); (**a**) 0.16 mL, (**b**) 0.33 mL, (**c**) 0.65 mL, and (**d**) 1.30 mL.

Wrinkled silica nanoparticles were synthesized in a bicontinuous microemulsion phase by hydrolysis and condensation of tetraethyl orthosilicate (TEOS). In the reaction mixture containing 30 mL distilled water, 30 mL cyclohexane, and 1.25 g of TEOS (6 mmol), various amounts of co-solvent with moderate hydrophobicity, namely 1-pentanol, were added in order to control the interwrinkle distances of the silica nanoparticles. Figure 2 shows the scanning electron microscope (SEM) images of the wrinkled silica nanoparticles with various interwrinkle distances, which were controlled by the different amounts of 1-pentanol added during the synthesis. For the preparation of nanoparticles shown in Fig. 2a–d, 0.16 mL, 0.33 mL, 0.65 mL, and 1.3 mL of 1-pentanol were introduced, respectively. The resulting morphologies of the wrinkled silica nanoparticles indicate that an increased amount of co-solvent widens the interwrinkle distances. During the synthesis of wrinkled silica nanoparticles, the reaction mixture forms a bicontinuous microemulsion phase (Winsor III system), in which 3 phases co-exist; oil (nonpolar solvent) at the top, bicontinuous microemulsion (composed of mostly surfactant and about equal amounts of water and oil) in the middle, and water (polar solvent) at the bottom^58^,^59^. When alcohols with long alkyl chain such as 1-pentanol are introduced into the reaction mixture, volume fraction of oil in the microemulsion phase increases. This results in the change in phase behavior toward Winsor II system, where water-in-oil microemulsion and oil layer exist together. Therefore, as the amount of added 1-pentanol increases, portion of oil phases in the reaction mixture gets higher. Since the oil to water ratio has a rough positive correlation to interwrinkle distances according to the previous report^58^, the observations on the morphologies of wrinkled silica nanoparticles seem to be reasonable. Among the 4 synthesis conditions, the 0.16 mL and 0.65 mL of 1-pentanol added cases were selected for the investigations on the effects of interwrinkle distance on scattering properties in visible light region. That is, conditions resulting in a siginificant difference in wrinkled morphology but maintaining the overall spherical structure of the particles were chosen. Wrinkled silica nanoparticles synthesized by the former and the latter experimental conditions were named as narrowly wrinkled silica nanoparticles (NWSNs) and widely wrinkled silica nanoparticles (WWSNs), respectively.

For the formation of TiO_2_ shell on the silica nanoparticle surface, hydrolysis and condensation of Ti(OBu)_4_ on silica surface were performed. The concentration of Ti(OBu)_4_ was varied for the formation of TiO_2_ shell with different thicknesses. Supplementary Fig. S1 shows the SEM images of NWSNs (Fig. S1a) and TiO_2_ coated NWSNs prepared with different amount of added Ti(OBu)_4_ solution (diluted in the mixed solution of ethanol and acetonitrile) to the reaction mixture (total volume: 220 mL); 0.5 mL (Fig. S1b), 1.0 mL (Fig. S1c), and 2.0 mL (Fig. S1d). As the amount of Ti(OBu)_4_ increased, TiO_2_ shell got thicker and completely filled the wrinkles of NWSNs in the case where 2.0 mL of Ti(OBu)_4_ solution was used (Fig. S1d). Considering that the target application of this silica/titania scattering particles is DSCs, thicker TiO_2_ shell is preferred for the enhanced electron transport. Since the conduction band position of silica is close to the vacuum level, conduction band electrons in TiO_2_ lattice cannot move into the silica but are only able to diffuse within TiO_2_, and thus thicker shells provide wider electron pathways with lower resistance. Meanwhile, in the aspects of surface area and hierarchical morphology of wrinkled structure, thin TiO_2_ shell is preferred. Therefore, TiO_2_ formations on the wrinkled silica by adding 1.0 mL of Ti(OBu)_4_ solution were selected for further investigation, because this condition gives relatively thick TiO_2_ shell without damaging the overall wrinkled structure significantly.

**Figure 3:**
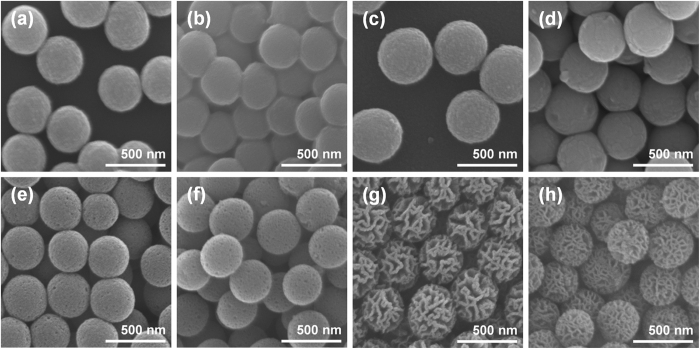
SEM images of scattering particles before (**a**,**c**,**e**,**g**) and after (**b**,**d**,**f**,**h**) heat treatment at 500 °C in air; (**a**,**b**) SSTNs, (**c**,**d**) STNs, (**e**,**f**) NWSTNs, and (**g**,**h**) WWSTNs.

Then, the effects of thermal annealing on the morphologies of the nanoparticles were investigated, because high temperature heat treatment is inevitable for the preparation of photoelectrode for DSCs in general. In order to compare the scattering abilities of wrinkled silica/titania nanoparticles with those of conventional scatterers, spherical silica/titania and titania nanoparticles of dimensions similar to the wrinkled silica/titania nanoparticle were prepared by hydrolysis and condensation of alkoxide precursors. SEM images in Fig. 3 show the morphologies of spherical silica/titania nanoparticles (SSTNs; Fig. 3a,b), spherical titania nanoparticles (STNs; Fig. 3c,d), narrowly wrinkled silica/titania nanoparticles (NWSTNs; Fig. 3e,f), and widely wrinkled silica/titania nanoparticles (WWSTNs; Fig. 3g,h) before (left side of Fig. 3) and after (right side of Fig. 3) heat treatment at 500 °C for 4 h in air. For all cases, there was no significant change in morphologies of the nanoparticles, indicating that the particles are sufficiently stable during the heat treatment.

**Figure 4:**
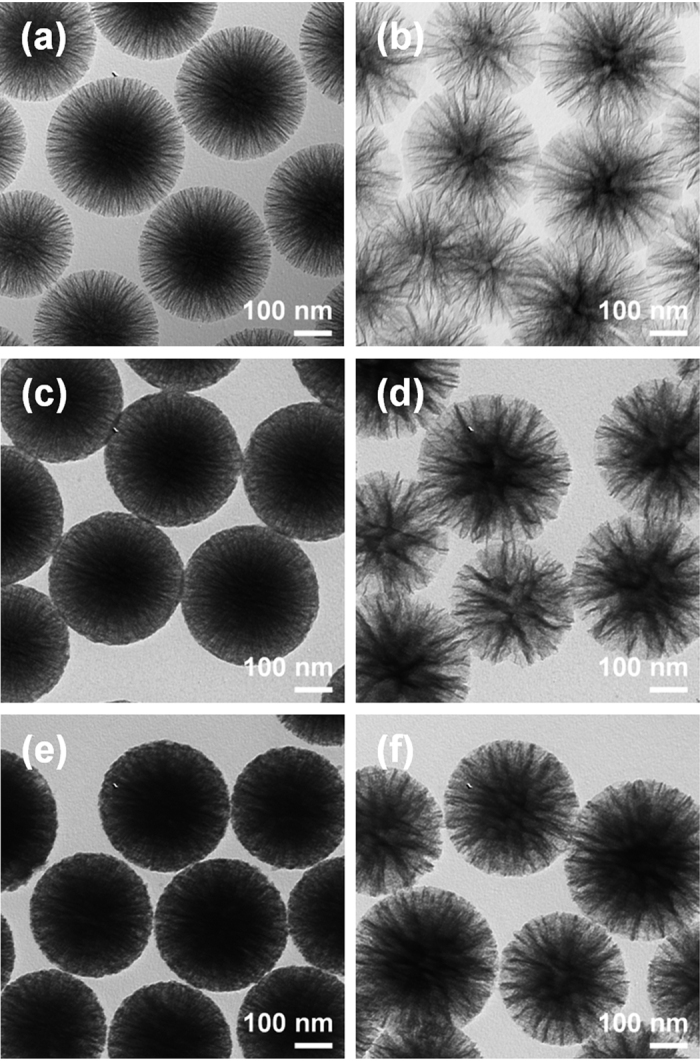
TEM images of (**a**) NWSNs, (**b**) WWSNs, (**c**,**e**) NWSTNs, and (**d**,**f**) WWSTNs (**c**,**d**) before and (**e**,**f**) after heat treatment at 500 °C in air.

**Figure 5:**
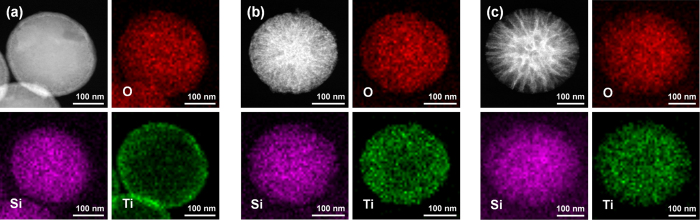
STEM images and corresponding elemental O, Si, and Ti EDS maps of (**a**) SSTNs, (**b**) NWSTNs, and (**c**) WWSTNs.

Moreover, the effects of TiO_2_ shell coating and heat treatment were confirmed by additional characterization based on transmission electron microscope (TEM) analyses (Fig. 4). Figure 4a–f show the morphologies of the NWSNs, WWSNs, NWSTNs, WWSTNs, thermally annealed NWSTNs, and WWSTNs after heat treatment, respectively. The darker images indicate that more dense structures were obtained after TiO_2_ coating, but there were no notable changes to the wrinkled morphology of the particles after heat treatment. Therfore, from these results, it is clear that the effect of TiO_2_ coatings on silica nanoparticles and that of heat treatment on the wrinkled structure is negligible. Figure 5 shows the scanning transmission electron microscope (STEM) images of SSTN (Fig. 5a), NWSTN (Fig. 5b), WWSTN (Fig. 5c) and corresponding elemental EDS mapping results on O, Si, and Ti. The STEM images and EDS maps clearly present that the TiO_2_ shells on silica surface are very uniform and do not affect the initial morphology of the silica nanoparticles. Moreover, cross-sections of silica/titania nanoparticles were directly observed by obtaining SEM images after focused ion beam (FIB) milling, as displayed in Supplementary Fig. S2.

### Physical properties and spectral characteristics

**Figure 6:**
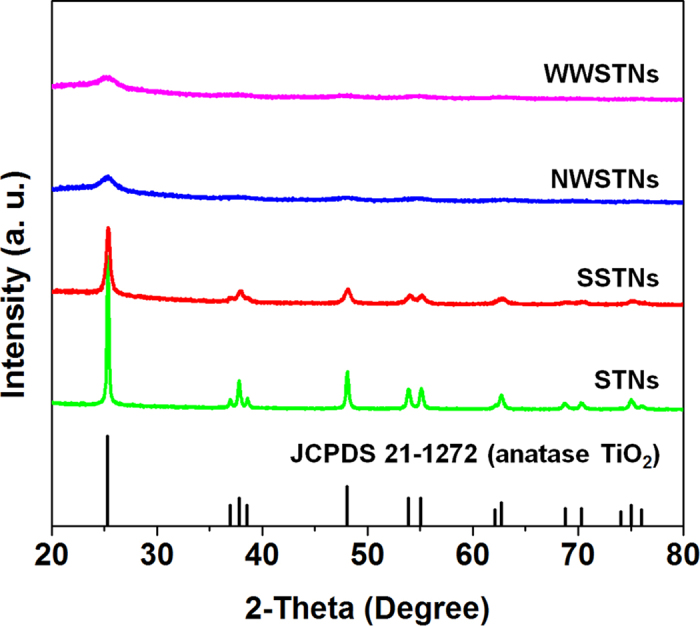
XRD patterns of WWSTNs, NWSTNs, SSTNs, and STNs after 500 °C heat treatment in air.

Figure 6 shows the XRD patterns of the WWSTNs, NWSTNs, SSTNs, and STNs. In all particles, anatase TiO_2_ peaks were apparent (JCPDS 21-1272) though the TiO_2_ signals in wrinkled nanoparticles were very broad. This indicates that the crystallite size of TiO_2_ shells in WWSTNs and NWSTNs are very small. In contrast, TiO_2_ in SSTNs showed relatively sharper anatase TiO_2_ peaks. The average grain sizes of anatase TiO_2_ in WWSTNs, NWSTNs, SSTNs, and STNs were calculated by (101) peak using Scherrer’s equation, and the results were 3.90 nm, 4.05 nm, 16.42 nm, and 34.66 nm, respectively, as summarized in Table 1. The differences between the crystallite sizes in each nanoparticle imply that the existence and increasing surface area of amorphous silica impede the growth of large anatase TiO_2_ grains. The presence of amorphous silica in SSTNs, NWSTNs, and WWSTNs were observed by the broad peak at 2-theta position of around 20 to 30 degrees (JCPDS 42-0005).

In DSCs, the surface area of the photoanode is one of the most critical factors for high performance. Because light-absorbing dye molecules adsorb as monolayers on the oxide semiconductor surface, larger surface area is advantageous. Therefore, the specific surface areas of the synthesized scattering particles were characterized and compared with that of ~20 nm sized commercial TiO_2_ nanoparticles (P25, Degussa). Supplementary Fig. S3 shows the N_2_ adsorption and desorption isotherms of P25, SSTNs, STNs, NWSTNs, WWSTNs, NWSNs, and WWSNs. The BET surface areas of these nanoparticles were calculated based on these results, and the values are displayed in Table 1 and Table S1. As expected from the analyses on their porous morphologies, wrinkled structures (>400 m^2^/g) provided more than 20 times larger surface area compared to the spherical particles (<20 m^2^/g). By comparing the specific surface areas of wrinkled silica nanoparticles before and after TiO_2_ coating, it was verified that TiO_2_ shell lowered the surface area to around 2/3 of the original value. Pore volumes of wrinkled silica and silica/titania nanoparticles were also measured by Barret-Joyner-Halenda (BJH) analysis, and significant decreases in pore volume (to around 1/2) after TiO_2_ shell deposition were observed, as displayed in Table 1 and Table S1. Combining the changes in specific surface areas and pore volumes during the deposition of titania, together with the alterations in pore size distributions (Supplementary Fig. S4), it is clear that thin TiO_2_ shell has been successfully formed within the mesopores larger than 5 nm, without damaging the original wrinkled nanostructure.

Interestingly, interwrinkle distances had no significant effect on the surface area. It was noteworthy to find that the difference between the surface areas of NWSTNs and WWSTNs was 0.31%, and that of NWSNs and WWSNs was 0.56%. These negligible differences verify that the synthesizing method proposed in this study – altering the amount of added co-solvent – enables the tailoring of interwrinkle distances without varying the specific surface area. Considering that the large surface area was maintained even after the deposition of titania shell, we could notice that our method is effective for the preparation of wrinkled nanostructure with finely controlled morphology and surface area for a wide range of applications. Meanwhile, there was significant gap between the surface area of SSTNs and STNs, which is ascribable to the different atomic weight of the Si and Ti. The atomic weight of Si is 28.086 g/mol, while that of Ti (47.867 g/mol) is significantly larger. This indicates that when the same weight of SSTNs and STNs are present, there is larger numbers of SSTNs compared to STNs, resulting in a larger surface area per unit mass.

**Figure 7:**
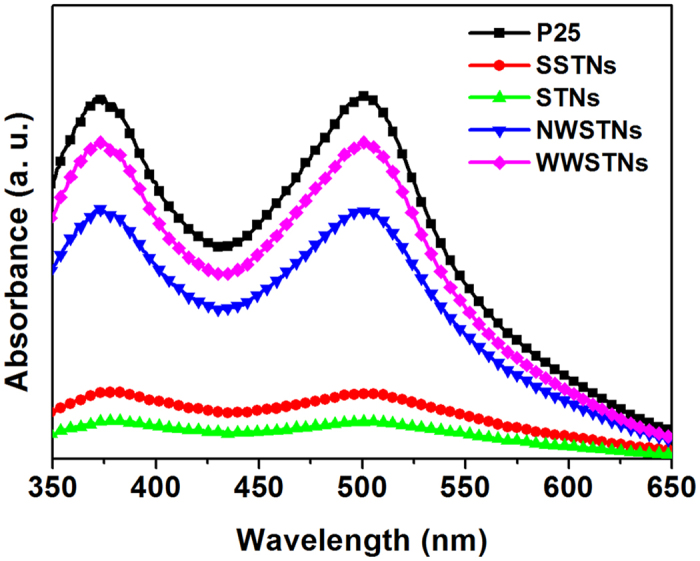
Absorbance spectra of N719 dye molecules detached from P25 and various scattering particles in 1 M NaOH solution.

Moreover, effective surface of the nanoparticles were compared by measuring the amount of adsorbed dye molecules on the surface of the particles of equal mass. The particles were immersed in ethanolic solution containing 0.5 mM N719 dye for 48 h at 30 °C, and then the dye molecules were detached by using 1 M NaOH solution. Figure 7 shows the absorbance spectra of N719 dye molecules detached from 10 mg of P25, SSTNs, STNs, NWSTNs, and WWSTNs, and Table 1 displays the loaded dye amounts on the oxide surface calculated by the extinction coefficient reported elsewhere^61^,^62^. Since the size of N719 dye molecule is much larger than N_2_ gas, trend of dye-loading amounts was different from that of BET surface areas. P25 had largest effective surface area (6.01 × 10^−5^ mol/g), and those of the wrinkled silica/titania nanoparticles were slightly lower; 4.26 × 10^−5^ mol/g for NWSTNs and 5.23 × 10^−5^ mol/g for WWSTNs. To our surprise, dye-loadings on wrinkled silica/titania nanoparticles were less than 10 times larger compared to those of spherical nanoparticles (1.33 × 10^−5^ mol/g for SSTNs and 0.77 × 10^−5^ mol/g for STNs). Unlike N_2_ molecules, relatively large N719 dye molecules seem to have been unable to move into the small pores in the wrinkled structures, and this can be inferred from the results of NWSTNs and WWSTNs. Though the difference in the specific surface area of NWSTNs and WWSTNs was only 0.31%, about 20% rise in the number of loaded dye molecules was observed as the interwrinkle distance increased. In short, this result implies that it is difficult for dye molecules to diffuse into the narrow nanostructures located within the wrinkled nanoparticles, though larger interwrinkle distances enhance the mass transport of dyes by providing wider pathways for dye molecules to move into deeper parts of the wrinkled pores.

**Figure 8:**
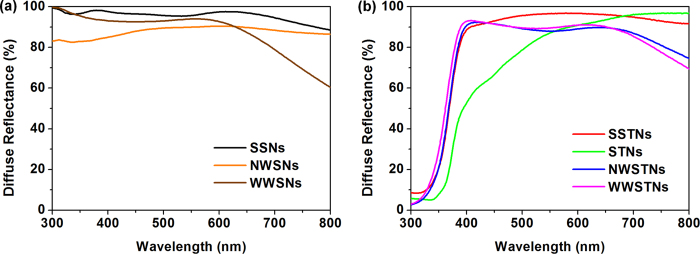
Diffuse reflectance spectra of (**a**) silica nanoparticles and (**b**) silica/titania and titania nanoparticles measured by using an integrating sphere.

Figure 8 shows the diffuse reflectance spectra of silica nanoparticles (Fig. 8a) and those of silica/titania and titania nanoparticlces (Fig. 8b). Among spherical and porous silica nanoparticles, spherical silica nanoparticles (SSNs) manifested largest reflectance in broadest spectral region, while the reflectivities of NWSNs and WWSNs were slightly lower than that. This result is mainly attributed to the lower density of wrinkled nanoparticles due to their porous morphologies^63^. Particles with porous structure contain empty space, wherein the air with low refractive index is present. In this case, the overall refractive index of wrinkled silica particles can be assumed by equation (1);


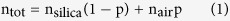


where n_tot_ is the overall refractive index of porous wrinkled silica, n_silica_ is the refractive index of bulk silica (n_silica_ > 1), n_air_ is the refractive index of air (n_air_ = 1), and p is the porosity of silica particles (0 < p < 1)^64^. Because the p value of wrinkled silica is significantly larger than SSNs for its porous structure, n_tot_ of wrinkled silica is smaller than that of SSNs, and this explains the smaller degree of refraction and less efficient light scattering in the case of wrinkled silica.

As we compared the diffuse reflectance spectra of NWSNs and WWSNs, an interesting observation on the dependence of light scattering properties on the interwrinkle distances of silica particles was made. Among the two types of wrinkled silica nanoparticles, the reflectance of NWSNs is higher than that of WWSNs in relatively larger wavelength region (above 630 nm). However, in lower wavelength region (below 630 nm), WWSNs exhibit higher magnitude of reflection than NWSNs do. These results imply that there is a relationship between the interwrinkle distance and the wavelength region showing high reflectance values. In general, spectral light scattering properties of nanoparticles are strongly related to the size of particles. The ideal relation between the size of a spherical scatterer and the wavelength was proposed as equation (2);


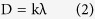


where D is the diameter of the scatterer, k is the constant, and λ is the wavelength of light^42^,^65^,^66^. Therefore, in optoelectronic devices including various types of photovoltaics, the size of scatterrer was often optimized for efficient light harvesting. Based on the effect of density and size of scatterers on the light-particle interactions, the different behavior of NWSNs and WWSNs can be expained. Despite of the similar particle sizes, WWSNs with lower density at the outermost part behave as particles with smaller diameter, reflecting smaller amount of the long-wavelength photons (>630 nm) compared to NWSNs. However, in the short-wavelength region (<630 nm), more intense light reflection was observed in the case of WWSNs, due to their well developed wrinkled structure which ranges from scores of a few to several hundreds of nanometers. These results indicate that the light scattering properties can be modified by controlled interwrinkle distances of wrinkled structures, without changes in the specific surface area as discussed above.

The trends in the diffuse reflectances of spherical and wrinkled nanoparticles were maintained after the formation of titania shell on the surface. Figure 8b shows the diffuse reflectance spectra of SSTNs, STNs, NWSTNs, and WWSTNs. Since TiO_2_ has band gap of around 3.2 eV, significant portion of photons with wavelengths below 380 nm were absorbed by the particles. However, all of the scattering nanoparticles were highly reflective in 400 – 800 nm wavelength range. SSTNs showed highest diffuse reflectance in broadest wavelength regions among silica/titania particles, and this can also be explained by the higher density and refractive index of spherical particles compared to those of nanoparticles with wrinkled morphology, as in the case of silica discussed above. Also, the reflectance spectra of NWSTNs and WWSTNs reveals that the correlation between the interwrinkle distances and the spectral light scattering behavior is still valid after the titania coating, though its degree was decreased. NWSTNs manifested higher reflectivity above 630 nm wavelength than WWSTNs, and the opposite trend was observed below 630 nm. Meanwhile, in the case of STNs, the reflectance was close to unity (>95%) at wavelength of 700 nm or higher, but it was reduced in the spectral region below 550 nm.

### Photovoltaic applications

For the preparation of DSCs with scatterers embedded within mesoporous TiO_2_ photoanode, scattering particles were dispersed in ethanol with certain amount of commercial paste containing 20.7 wt% of ~20 nm sized TiO_2_ nanoparticles (DSL 18NR-T, Dyesol)^33^. After harsh ultrasonication for uniform dispersion, ethanol in the mixture solution was evaporated, and viscous pastes containing 10 wt% of scattering nanoparticles in regard to the amount of original TiO_2_ content were obtained. The pastes containing SSTNs, STNs, NWSTNs, WWSTNs, or no scatterer were cast on FTO glass and were sintered in air at 500 °C for 30 min in order to remove solvent and organic binders, and also to enhance the connectivity between the nanoparticles.

Then we performed characterizations on scattering properties and measured dye-loadings on the fabricated electrodes in order to confirm the effectiveness of the light scattering nanoparticles in actual TiO_2_ photoanodes. As can be seen from Supplementary Fig. S5, wherein diffuse reflectance of TiO_2_ photoanodes containing 10 wt% of scatterers are displayed, substantial increases in light scattering beyond the absorption edge of TiO_2_ (~380 nm) were achieved. Also, it was notable to observe that the spectral responses from the photoanodes were in accordance with the reflectance spectra of spherical and wrinkled scattering particles shown in Fig. 8. Dye-sensitization of the photoelectrodes were carried out by immersing the electrodes into ethanolic N719 dye solution for 48 h, and the amount of loaded dye molecules were obtained by using the same method used in specific dye-loading measurements in the previous part of this paper. As can be seen from the results displayed in Table 2, the trend in dye-loadings well matched with the specific dye-loading amounts displayed in Table 1. From the agreements between the trends of bare scattering particles and photoelectrodes employing 10 wt% of scatterers in both light scattering and dye-loading, we could conclude that the characteristics of spherical and wrinkled nanoparticles would be valid in photovoltaic applications.

**Figure 9:**
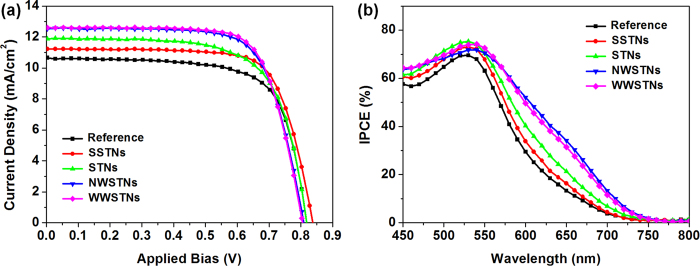
(**a**) *J-V* characteristics and (**b**) IPCE spectra of reference DSC and DSCs employing spherical or wrinkled scattering nanoparticles.

Figure 9a shows the photocurrent density (*J*)-voltage (*V*) characteristics of the DSCs employing the scattering nanoparticles, and the parameters obtained from the *J-V* curve representing the photovoltaic performances are displayed in Table 2. By investigating the short-circuit photocurrent density (*J*_sc_) values, magnitude of light scattering and surface area can be indirectly compared, because *J*_sc_ has strong positive correlation with light harvesting efficiency, which is related to the loaded dye amounts and efficient light scattering^7^. DSCs employing wrinkled silica/titania nanoparticles as scatterers showed largest *J*_sc_ values, and also the highest energy conversion efficiencies were observed in these cells. This is mainly attributed to the large surface area for dye-loading and efficient light scattering properties of wrinkled nanoparticles. As can be seen from the measured values in Table 2, the amounts of loaded dyes on the photoanode incorporating NWSTNs and WWSTNs are about 95% of that on the reference photoanode composed of only ~20 nm sized TiO_2_ nanoparticles despite their large particle size. Also, from the IPCE spectra in Fig. 9b, it is clear that the DSCs with wrinkled silica/titania particles exhibited largest external quantum efficiency in the wavelength region beyond 550 nm, verifying the excellent light scattering and harvesting properties of NWSTNs and WWSTNs. In addition, one noteworthy observation was made on IPCE results that the trend in spectral light scattering behaviors observed by the diffuse reflectance spectra are still valid in photovoltaic application. DSCs employing WWSTNs, which is more effective for the scattering of photons with shorter wavelengths that overlaps with the N719’s absorption region, manifested higher performance compared to those with NWSTNs. This indicates the usefulness of spectral modification of light-scattering wrinkled silica nanoparticles by altering the interwrinkle distances.

Given that the *J*_sc_ and *η* is higher in DSCs with SSTNs or STNs compared to the reference DSCs, it is clear that these spherical particles did scatter light to some extent and contributed in the enhanced light harvesting. However, DSCs employing SSTNs or STNs showed lower *J*_sc_ values compared to the case of wrinkled particles, mainly due to the fewer amounts of loaded dye molecules. Meanwhile, there is one more interesting observation from the *J-V* characteristics of the DSCs that the presence of silica cores irrespective to their morphology slightly increased the fill factor (*FF*) of DSCs. Since the similar behaviors were also reported in the cases of DSCs with scattering voids in mesoporous TiO_2_ layer^32^,^33^, this can be attributed to the reduced spaces for TiO_2_ conduction band electrons to travel through, because silica with its conduction band edge near vacuum level serves as insulator in DSCs. In this situation, larger portion of electron seem to diffuse and arrive at the current collector before charge recombination, and it can also be indirectly confirmed by the higher open-circuit voltage (*V*_oc_) of DSCs with SSTNs compared to those of DSCs with STNS or without any scattering particles, because *V*_oc_ is negatively correlated to the overall charge recombination rate^67^. However, *V*_oc_ values of DSCs employing wrinkled silica/titania nanoparticles were the lowest among all of the cells, and this is probably due to the recombination at the TiO_2_ shell on the silica wrinkles wherein large population of grain boundaries with defect sites are located, as verified by the XRD results. This can also be confirmed by lower *FF*s in DSCs with wrinkled scatterers than in that with SSTNs, though the gap is very small.

**Figure 10:**
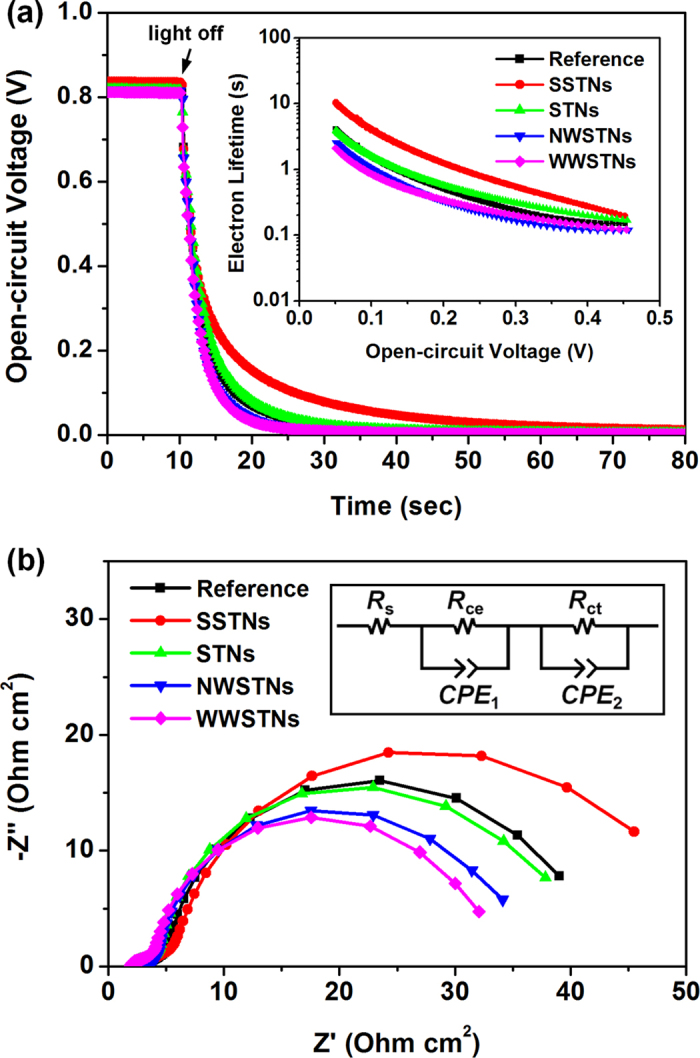
(**a**) OCVD curves of DSC and DSCs employing spherical or wrinkled scattering nanoparticles. The electron lifetimes calculated from the OCVD results are displayed in the inset. (**b**) Electrochemical impedance spectra of reference DSCs with or without scattering particles.

In order to further understand the differences in photovoltaic parameters, especially *V*_oc_, open-circuit voltage decay (OCVD) measruements were performed. Figure 10a shows the OCVD results and electron lifetime (*τ*_e_) calculated according to the following equation (3);


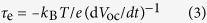


where *k*_B_ is the Boltzmann constant, *T* is the absolute temperature, and *e* is the positive elementary charge^68^,^69^. Since a larger *τ*_e_ indicates a lower charge recombination rate, comparison between the *τ*_e_ of DSCs with SSTNs and those with STNs clearly shows that the presence of silica core in scattering particles alleviates back-reaction. The reference DSCs exhibited slightly lower *τ*_e_ than the DSCs with STNs, and this can be attributed to the relatively large crystallite sizes of STNs (see Table 1 for the exact value), which makes electrons to encounter a smaller number of defect sites in grain boundaries. The lowest *τ*_e_ values were observed in the cases of DSCs employing NWSTNs or WWSTNs, mainly due to the densely populated recombination centers caused by small crystallite sizes (see Table 1) in the TiO_2_ shell of the wrinkled scattering particles.

The results from the OCVD analyses were cross-checked by electrochemical impedance spectroscopy (EIS). Figure 10b shows the Nyquist plot of DSCs measured in dark condition with the forward bias of 0.7 V. Distinct differences were observable in higher frequency region (larger semicircle on the right side), wherein the responses from charge transfer kinetics at photoanode/electrolyte interfaces are collected^70^,^71^. We fitted the impedance spectra according to the equivalent circuit displayed in the inset, and obtained interfacial charge transfer resistance (*R*_ct_) at the photoanode/electrolyte interfaces and chemical capacitance (*C*_μ_, capacitance part of *CPE*_2_ in the circuit) of the photoelectrodes from the second semicircle. The exact values of the fitted parameters are displayed in Table 3. Since *R*_ct_ shows the magnitude of hinderance for electron transfer at TiO_2_/electrolyte interface^72^, a larger *R*_ct_ value may indicate that the charge recombination reaction is suppressed to some extent. *C*_μ_ stands for the degree of accumulated electrons in TiO_2_^72^. In DSCs with or without scattering nanoparticles, the trend in *R*_ct_ and *C*_μ_ well agreed with each other, in a descending order of SSTNs, STNs, Reference, NWSTNs, and WWSTNs. This result seems reasonable, because a reduced charge recombination rate would contribute to a larger population of conduction band electrons in TiO_2_. Also, the trends in *R*_ct_ and *C*_μ_ values well matched with the *V*_oc_ of the cells. Therefore, we could conclude that the discussion performed on *V*_oc_ in the previous part was plausible. Moreover, in order to compare the information from EIS with that from OCVD analysis, we calculated mean electron lifetime (*τ*_n_) based on the equation (4) as follows^72^;


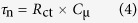


and the values are displayed in Table 3. As could have been expected, the trend in *τ*_n_ was the same to those in the case of *R*_ct_ and *C*_μ_. Moreover, we could confirm that the electron lifetimes obtained from OCVD and EIS have the same tendency, in the descending order mentioned above.

Combination of the results from *J-V*, OCVD, and EIS analyses provide clear understanding on the origin of differences in photovoltaic parameters of DSCs employing various scattering particles, especially on *V*_oc_ which is closely related to electron lifetimes governed by charge recombination kinetics. This means that our wrinkled silica/titania nanoparticles have limitations originated from the relatively fast charge recombinations. However, since these scattering particles were verified to be highly effective for improving the performances of DSCs (due to the large amount of dye-loading and favorable light scattering properties), the investigations based on the electron lifetime indicate that there are possibilities for further improvements by tailoring the crystallinity of TiO_2_ shells on wrinkled silica nanoparticles, which would suppress back-reactions and maximize the energy conversion efficiency of DSCs.

## Discussion

In summary, wrinkled silica nanoparticles with tunable interwrinkle distances were prepared by altering the amounts of added hydrophobic co-solvent, namely 1-pentanol in this research, to the bicontinuous microemulsion phase (Winsor III system) containing TEOS (reactant), CTAB (surfactant), cyclohexane (nonpolar solvent), and water (polar solvent). These wrinkled silica particles were coated with TiO_2_ shell and then were used as scatterers in the photoanodes of DSCs. Compared to the conventional sphere-shaped scattering nanoparticles, wrinkled silica/titania displayed superior performance in DSCs due to their large surface area for dye-loading. Moreover, observations on the relationship between interwrinkle distances of wrinkled nanoparticles and spectral reflectivity were made, and the effectiveness of this approach was verified in DSC applications. Considering the simplicity and reliability of the method for interwrinkle distance control, the spectral engineering of the scattering particles based on wrinkled silica is anticipated to be very useful in various applications which require efficient utilization of photons.

## Methods

### Synthesis of wrinkled silica/titania nanoparticles

All of the reagents used in this study were purchased from Sigma-Aldrich, or otherwise stated, and were used in the experiments without further purification. Wrinkled silica nanoparticles were synthesized in a bicontinuous microemulsion phase containing water and oil by hydrolysis and condensation of TEOS (TCI) using CTAB as surfactant. 1 g of CTAB and 600 mg of urea (Samchun, Korea) were dissolved in 30 mL of distilled water, and then various amounts of 1-pentanol in 30 mL Cyclohexane (Samchun, Korea) was added, which was followed by 30 min stirring of the mixture solution at room temperature. Under vigorous stirring, 1.25 g (6 mmol) of TEOS was added dropwise to the mixture solution, and the reaction temperature was raised to 70 °C immediately and maintained for 16 h. The white products were washed with ethanol by centrifugation, which was conducted three times at 13,000 rpm for 10 min each, and the products were re-dispersed in ethanol. In order to remove the remaining CTAB in the product, 10% HCl aqueous solution (1:10 vol. ratio to ethanol) was added and the mixture was stirred overnight. Then the products were purified by centrifugation, and 200 mg of them were dispersed in 200 mL of mixed solution of acetonitrile and ethanol of which composition were 1:1 in volumetric ratio, and 2 mL of ammonia solution (28%, Samchun, Korea) was added. Ti(OBu)_4_ (0.5~2 mL) was diluted in the mixed solution of ethanol and acetonitrile (1:1 vol. ratio) to have a total volume of 20 mL and was injected. The temperature of the solution was maintained at 25 °C for 2 h and the final product was obtained by three times of centrifugation (13,000 rpm for 10 min each).

### Synthesis of spherical scattering nanoparticles

SSTNs and STNs were prepared based on seed growth method. For the synthesis of SSTNs, silica cores were prepared by Stöber method. 1.2 mL of TEOS was dissolved in mixture of 1-propanol and 1-butanol (1:1 vol. ratio), and 6 mL of ammonia solution, and 2 mL of distilled water were added. After 6 h, white products were formed, and they were dispersed in ethanol after purification by centrifugation. Then the titania shell was coated on silica particle by the same method used for the synthesis of wrinkled silica/titania particles. For the preparation of the STNs, 1.5 mL of Ti(OBu)_4_ dissolved in 60 mL of mixture solution (acetonitrile and ethanol by 1:1 vol. ratio) was added to the 200 mL mixture solution containing 2 mL of ammonia solution. After 2 h, ammonia solution (0.5 mL) and Ti(OBu)_4_ (1.0 mL) in the mixed acetonitrile and ethanol solution (1:1 vol.) were added to increase the size of titania particles. The products were purified by centrifugation after 2 more hours of reaction.

### Preparation of dye-sensitized solar cells

For the fabrication of mesocopic TiO_2_ photoanode with embedded scattering particles, commercial paste containing ~20 nm sized TiO_2_ nanoparticles (DSL 18NR-T, Dyesol) was dispersed in ethanol. Based on the measurements of TiO_2_ content in the paste (20.7 wt%) by Grätzel and his colleagues^33^, wrinkled and spherical silica/titania nanoparticles and spherical titania nanoparticles were mixed with the ethanolic TiO_2_ paste solution with the amount of scatterer to be 10 wt% after removing solvents and organic binders. After 5 min (2 sec on - 2 sec off for 10 min) of ultrasonication for uniform dispersion of scattering nanoparticles, ethanol in the mixture solution was removed by rotary evaporation, resulting in a viscous paste as a final product. For the exact evaluation of embedded scattering particles, paste without scatterers was prepared by dispersion of commercial TiO_2_ paste in ethanol followed by evaporation of ethanol. Then the paste was cast on the FTO glass with TiO_2_ blocking layer by doctor blading method followed by heat treatment at 500 °C in air. TiCl_4_-post treatment^73^,^74^ was avoided in this study in order not to make changes to the TiO_2_ shell in the silica/titania nanoparticles. The prepared electrodes were immersed into an ethanolic solution containing 0.5 mM N719 dye (Ru 535-bisTBA, Solaronix) solution for 48 h at 30 °C for the positioning of dye molecules on the semiconductor oxide surface. Counter electrodes were prepared by spin coating of 50 mM H_2_PtCl_6_ containing 2-propanol solution on FTO glass and following thermal treatment at 400 °C for 20 min in air. For the cell assembly, 50 μm thick thermoplastic sealants (Surlyn, DuPont) were used, and the electrolyte containing I_3_^−^/I^−^ redox couple was injected through the pre-drilled holes. The exact composition of the electrolyte was 0.6 M 1-butyl-3-methylimidazolium iodide, 30 mM I_2_, 0.1 M guanidinium thiocyanate, and 0.5 M 4-*tert*-butylpyridine in a mixture solution of acetonitrile and valeronitrile with vol. ratio of 85:15.

### Physicochemical characterizations and photoelectrochemical measurements

The morphologies of synthesized particles were characterized by using a scanning electron microscope (SEM; Hitachi S-4300) and a transmission electron microscope (TEM; Hitachi-7400). Cross-sectional SEM images were obatined by Carl Zeiss AURIGA after FIB milling, and more detailed TEM images and elemental analyses were performed by using an analytical TEM (FEI Tecnai F20) equipped with an energy dispersive spectroscopy (EDS) system. UV-Vis absorbance spectra were obtained with Beckman DU 650 spectrophotometer, and diffuse reflectances were measured by using Jasco V-670 spectrophotometer with an integrating sphere. X-ray diffraction (XRD) patterns were obtained by using Rigaku D/MAX 2500 V with Cu Kα radiation source, and Brunauer-Emmett-Teller (BET) analyses were performed with Micrometrics ASAP 2420. Photoelectrochemical evaluations of DSCs were performed by obtaining photocurrent density-voltage curve under incident AM 1.5G light (intensity: 100 mW/cm^2^) by using a solar simulator (XIL model 05A50KS source measure units equipped with an AM 1.5G filter) and a potentiostat (Solartron 1480). Incident photon-to-current efficiency (IPCE) spectra were obtained by McScience K3100, and electrochemical impedance spectra were measured by using Zahner Zennium with the sinusodial perturbation of 10 mV.

## Additional Information

**How to cite this article**: Kang, J. S. *et al*. Wrinkled silica/titania nanoparticles with tunable interwrinkle distances for efficient utilization of photons in dye-sensitized solar cells. *Sci. Rep.* **6**, 30829; doi: 10.1038/srep30829 (2016).

## Supplementary Material

Supplementary Information

## Figures and Tables

**Figure 1:**
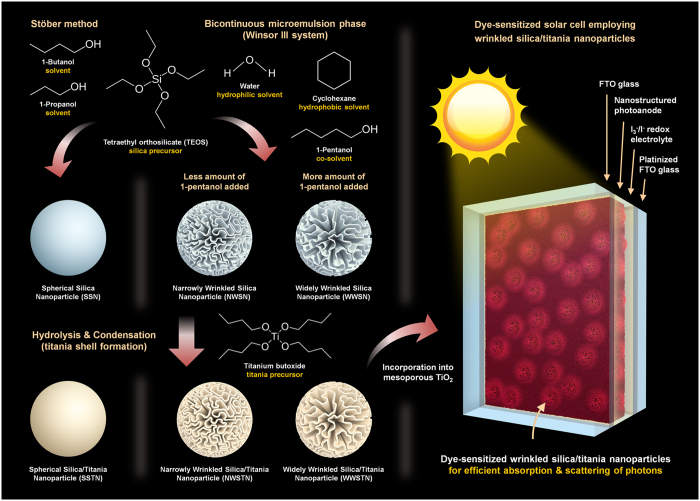
Schematic image displaying the synthesis of silica/titania nanoparticles and their photovoltaic application.

**Table 1:** Average crystallite sizes, BET surface areas, pore volumes, and specific N719 dye-loading amounts of P25 TiO_2_ nanoparticles, spherical scattering particles, and wrinkled scatterers.

	Crystallite Size of anatase TiO_2_ (nm)	BET Surface Area (m^2^/g)	Pore Volume^a^ (cm^3^/g)	Specific Dye-loading (10^−5^ mol/g)
P25	25^b^	40.12	0.113	6.01
SSTNs	16.42	17.29	0.043	1.33
STNs	34.66	13.52	0.026	0.77
NWSTNs	4.05	417.70	0.757	4.23
WWSTNs	3.90	416.39	1.252	5.23

^a^Pore volume at P/P0 = 0.99.

^b^This value is adapted from ref. 75.

**Table 2:** N719 dye-loadings on photoanodes and *J*-*V* characteristics of the reference DSC without scatterer and DSCs employing spherical and wrinkled scatterering particles.

	Dye-loading (10^−7^ mol/cm^2^)	****Voc** (V)	****J_sc_** (mA/cm^2^)	Fill Factor (%)	Efficiency (%)
Reference	1.423	0.819	10.7	70.4	6.17
SSTNs	1.212	0.838	11.2	73.1	6.86
STNs	1.179	0.819	11.9	68.4	6.67
NWSTNs	1.351	0.809	12.5	71.4	7.22
WWSTNs	1.364	0.806	12.6	72.4	7.35

**Table 3:** Parameters obtained from the EIS analysis of DSCs with or without scattering particles by fitting the EIS spectra in Fig. 10b according to the equivalent circuit displayed in the inset of Fig. 10b.

	***R***_ct_ (Ω cm^2^)	***C***_**μ**_ (μF/cm^2^)	***τ***_n_ (ms)
Reference	35.26	2051	72.32
SSTNs	43.67	2237	97.69
STNs	35.36	2108	74.54
NWSTNs	31.16	2050	63.88
WWSTNs	28.73	1942	55.79
