# Supplementary material for: Wrinkled silica/titania nanoparticles with tunable interwrinkle distances for efficient utilization of photons in dye-sensitized solar cells
Source: Sci Rep. 2016 Aug 4;6:30829. doi: 10.1038/srep30829 (PMC4973230; doi:10.1038/srep30829)
Supplement: Supplementary Information [file srep30829-s1.doc]

**Supplementary Information**

**Wrinkled silica/titania nanoparticles with tunable interwrinkle distances for efficient utilization of photons in dye-sensitized solar cells**

Jin Soo Kang^1,2^,*, Joohyun Lim^3^,*^,†^, Won-Yeop Rho^4^, Jin Kim^1,2^, Doo-Sik Moon^3^,

Juwon Jeong^1,2^, Dongwook Jung^3^, Jung-Woo Choi^1,2^, Jin-Kyu Lee^3^ & Yung-Eun Sung^1,2^

^1^Center for Nanoparticle Research, Institute for Basic Science (IBS), Seoul 151-742, Republic of Korea.

^2^School of Chemical and Biological Engineering, Seoul National University, Seoul 151-742, Republic of Korea.

^3^Department of Chemistry, Seoul National University, Seoul 151-742, Republic of Korea.

^4^Department of Bioscience and Biotechnology, Konkuk University, Seoul, 143-701, Republic of Korea.

*These authors contributed equally to this work.

^†^Present address: Department of Chemistry and Nanoscience, College of Natural Science, Ehwa Womans University, Seoul 120-750, Republic of Korea.

CORRESPONDING AUTHORS

Yung-Eun Sung (e-mail: ysung@snu.ac.kr) & Jin-Kyu Lee (e-mail: jinklee@snu.ac.kr)

**Table S1.** BET surface areas of wrinkled silica nanoparticles with different interwrinkle distances.

|  | BET Surface Area  (m^2^/g) | Pore Volume^a^  (cm^3^/g) |
| --- | --- | --- |
| NWSNs | 646.40 | 1.494 |
| WWSNs | 642.81 | 2.122 |

^a^Pore volume at P/P_0_ = 0.99.


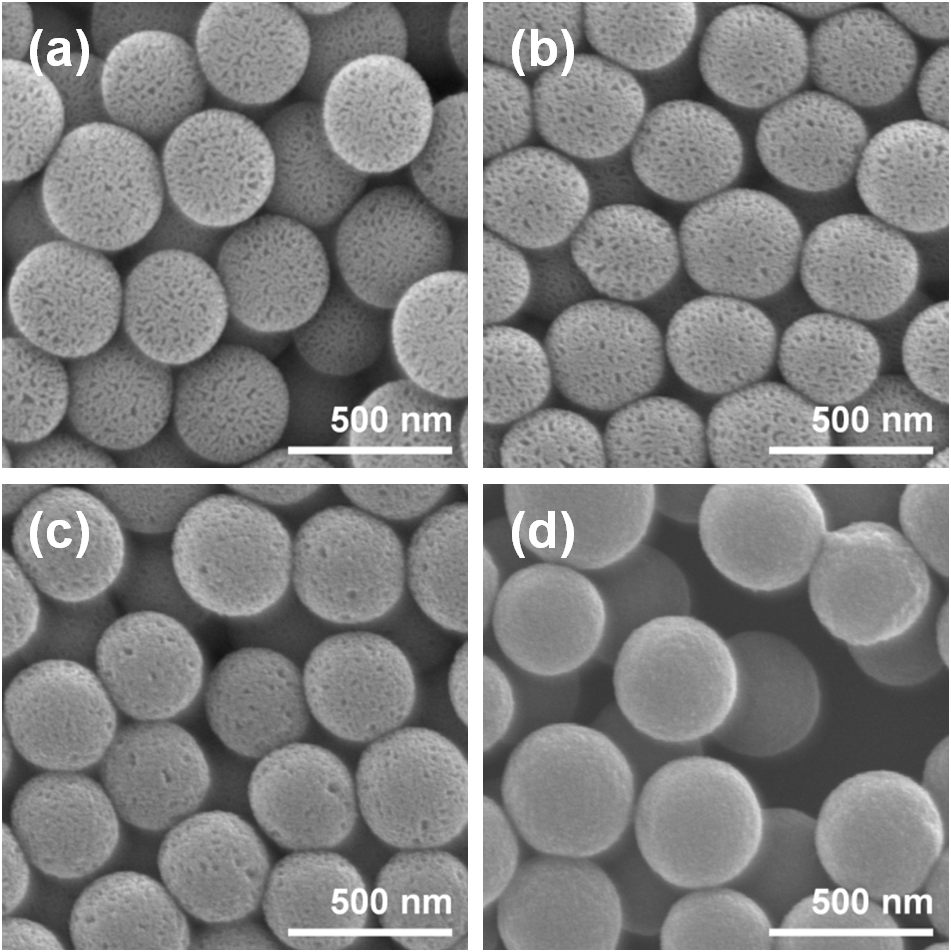


**Figure S1.** SEM images of narrowly wrinkled silica nanoparticles (a) and the same particles with TiO_2_ shell on the surface (b-d), of which thickness varied by different amount of TiO_2_ precursor; (b) 0.5 mL, (c) 1.0 mL, and (d) 2.0 mL.


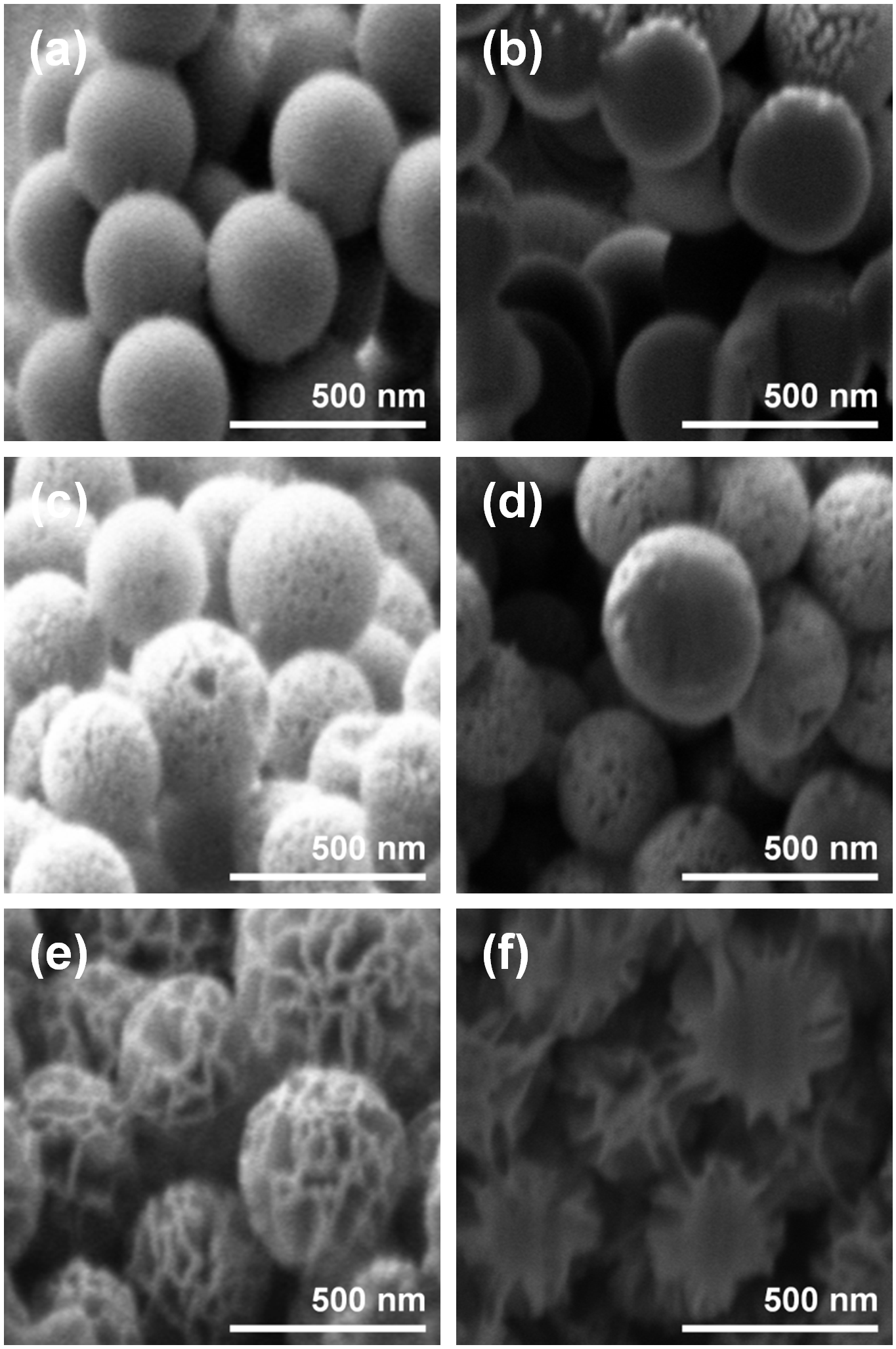


**Figure S2.** SEM images of (a,b) SSTNs, (c,d) NWSTNs, and (e,f) WWSTNs before (a,c,e) and after (b,d,f) focused ion beam (FIB) milling.


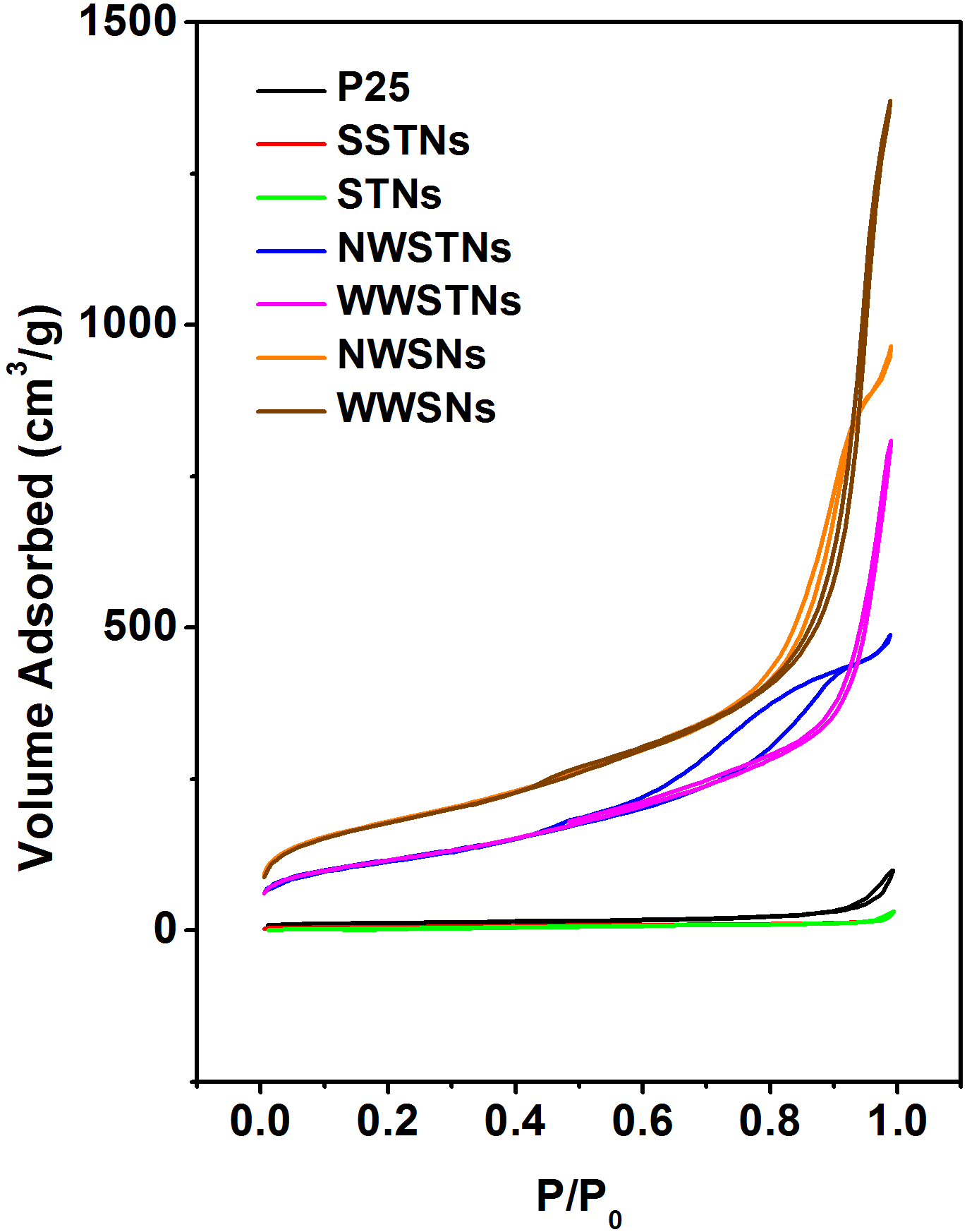


**Figure S3.** N_2_ adsorption-desorption isotherms of P25 TiO_2_ nanoparticles, spherical scattering nanoparticles, wrinkled silica/titania nanoparticles, and wrinkled silica nanoparticles.


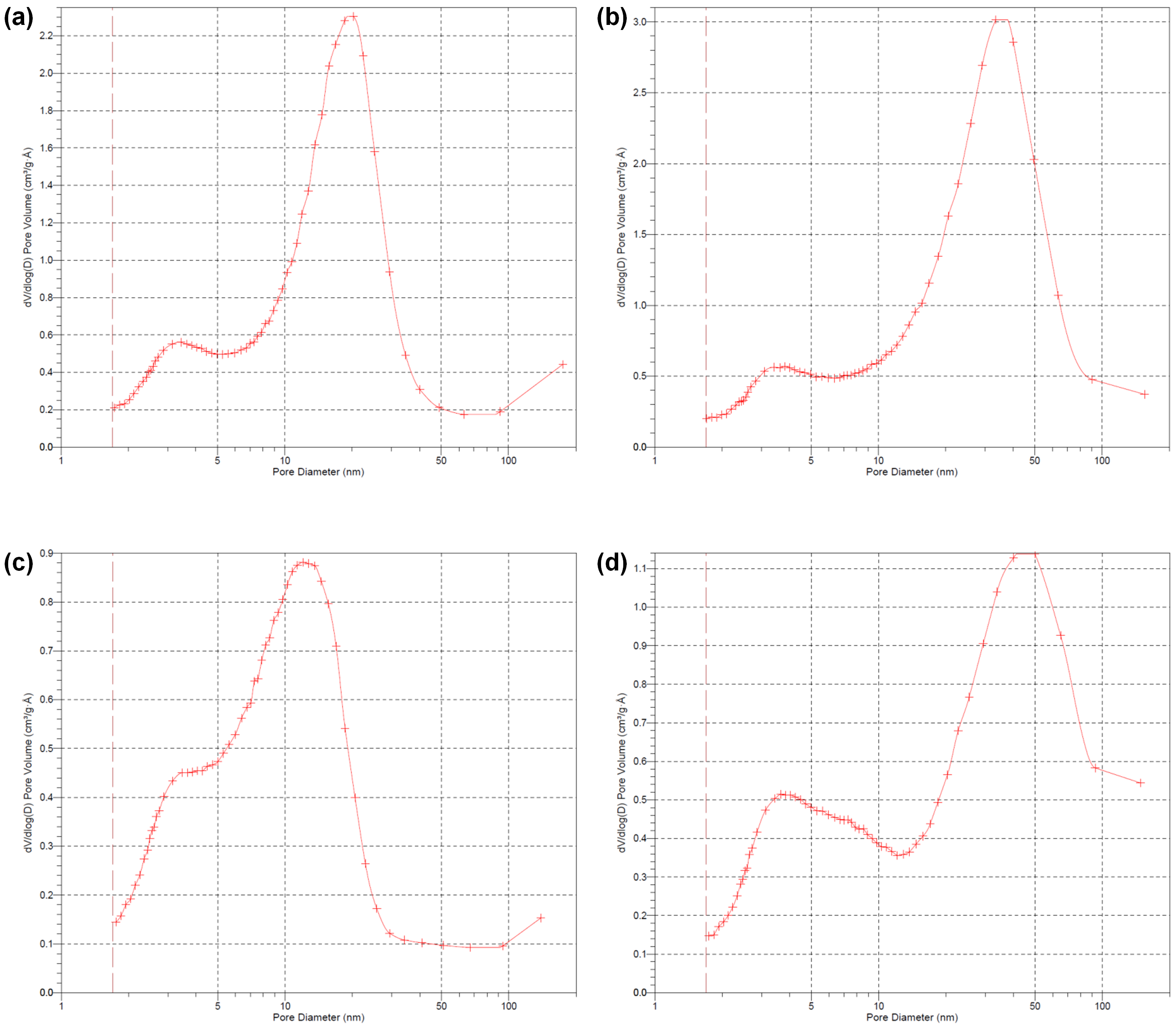


**Figure S4.** Pore size distribution of (a) NWSNs, (b) WWSNs, (c) NWSTNs, and (d) WWSTNs.


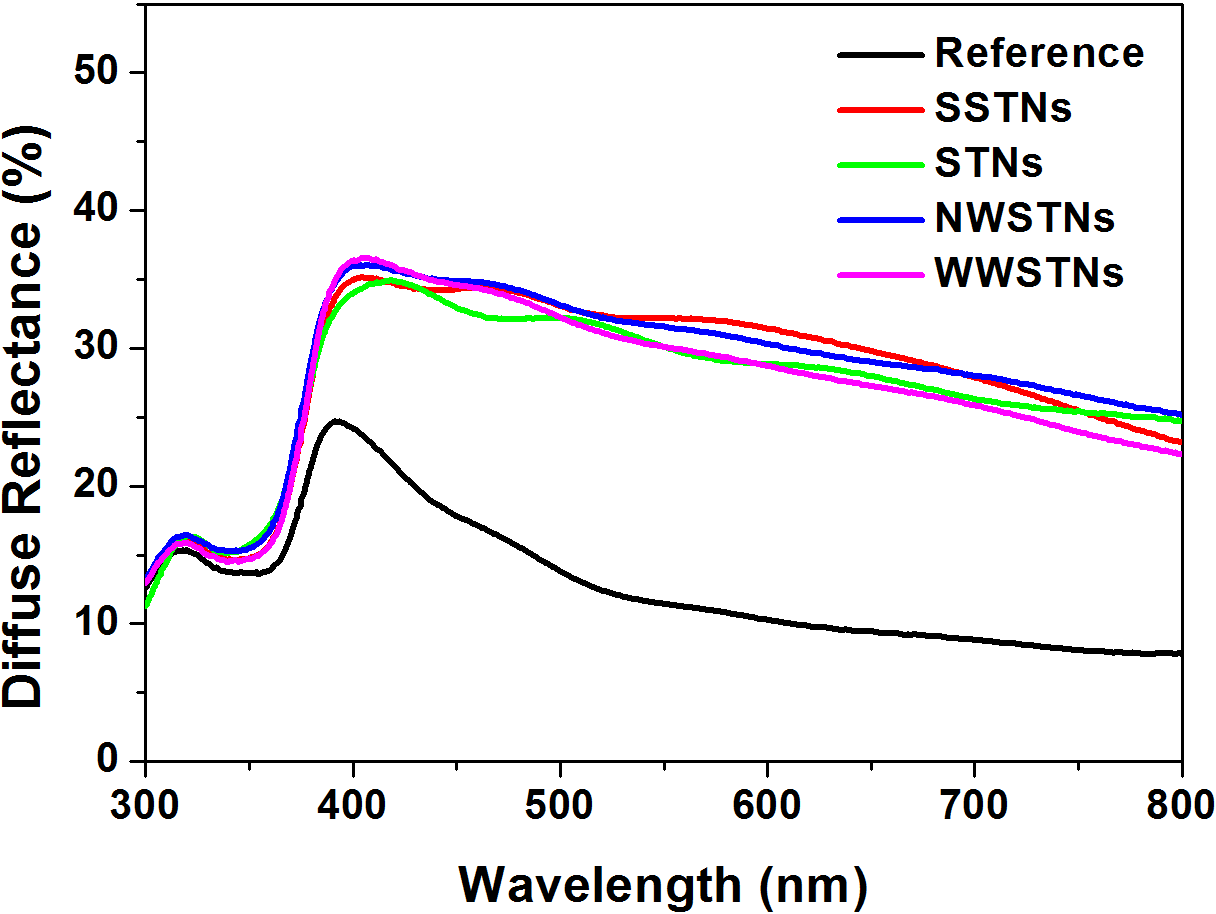


**Figure S5.** Diffuse reflectance spectra of reference and scattering particle-containing (10 wt%) TiO_2_ photoanodes without dye molecules adsorbed on the surface.
